# Coronary Embolism Causing STEMI and Complete Heart Block During PTMC in Pregnancy

**DOI:** 10.1016/j.jaccas.2026.108881

**Published:** 2026-06-13

**Authors:** Rajesh Vijayvergiya, Anirudh Mukherjee, Basant Kumar, Parminder Sharma

**Affiliations:** Department of Cardiology, Post Graduate Institute of Medical Education & Research, Chandigarh, India

**Keywords:** acute coronary syndrome, cardio-obstetrics, complete heart block, coronary embolism, percutaneous transvenous mitral commissurotomy, pregnancy, rheumatic mitral stenosis, ST-segment elevation myocardial infarction, stentless percutaneous coronary intervention, thrombectomy

## Abstract

**Background:**

Severe rheumatic mitral stenosis during pregnancy carries high maternal-fetal risk. Percutaneous transvenous mitral commissurotomy (PTMC) is the guideline-recommended therapy.

**Case Summary:**

A 33-year-old primigravida at 31 weeks' gestation with critical rheumatic mitral stenosis (mitral valve area: 0.47 cm^2^) and NYHA functional class III symptoms underwent urgent PTMC. Immediately after PTMC, she developed profound hypotension, complete heart block, and an inferior ST-segment elevation myocardial infarction (STEMI). Emergent coronary angiography revealed thrombotic occlusion of the mid right coronary artery with TIMI flow grade 0. Prompt mechanical thrombectomy restored TIMI flow grade 3. The patient delivered vaginally at 36 weeks, and both mother and neonate had favorable outcomes.

**Discussion:**

This case highlights the importance of early recognition of STEMI and demonstrates that a stentless percutaneous coronary intervention can successfully restore coronary flow.

**Take-Home Message:**

Immediate recognition of thrombotic occlusion of coronary artery during PTMC is essential for an emergent tailored stentless percutaneous coronary intervention during pregnancy.

## Graphical Abstract

**Figure:**
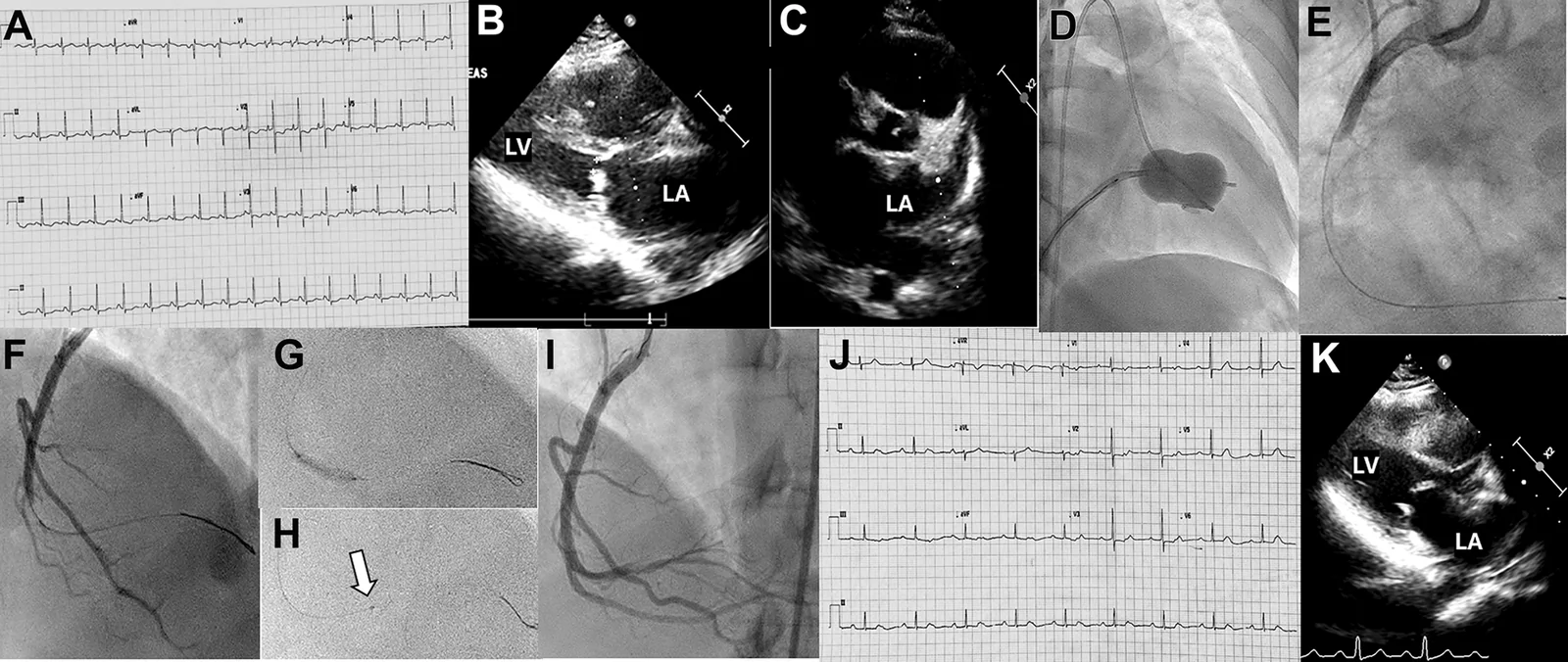

**Visual Summary:**
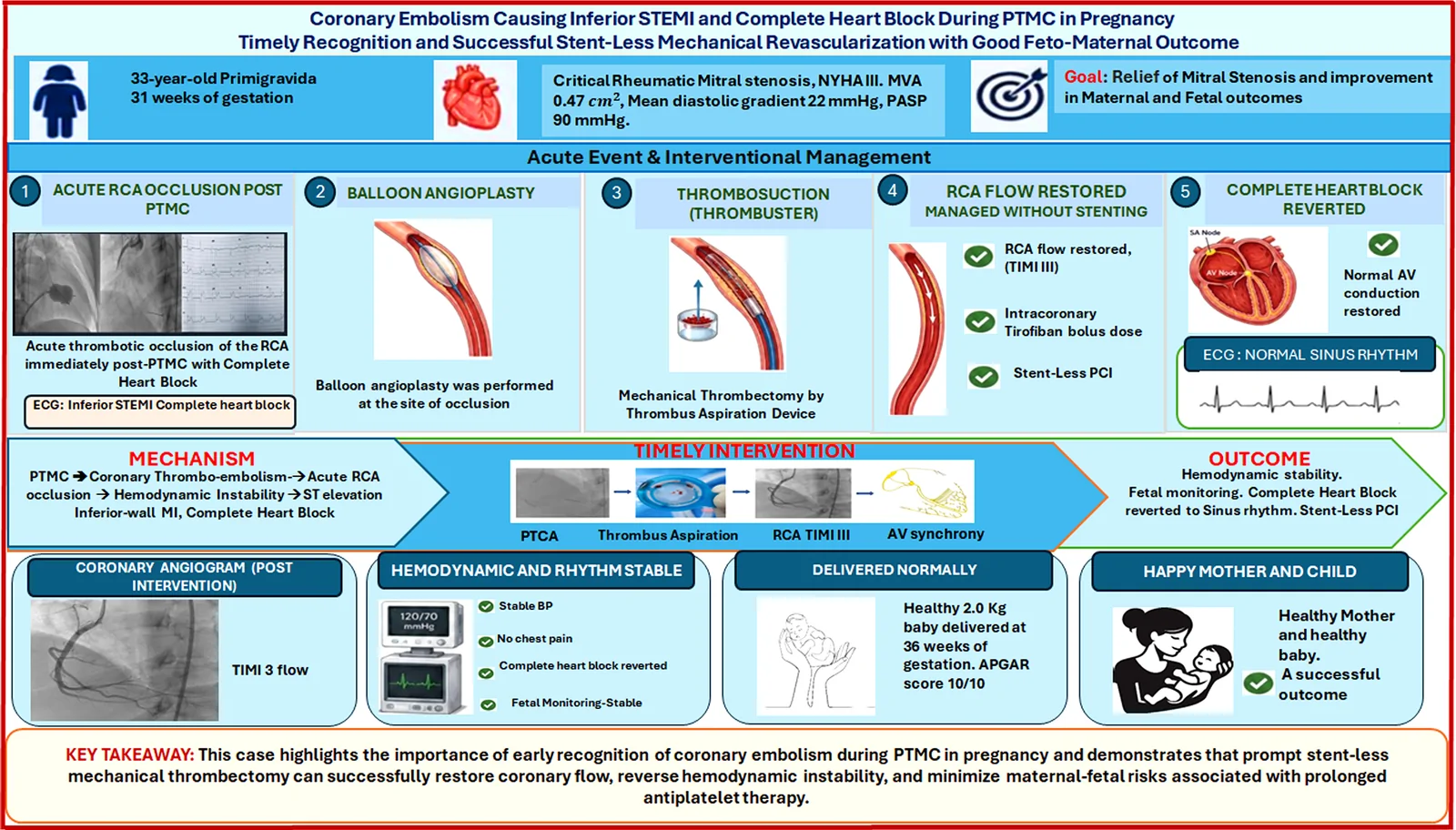
Coronary Embolism Causing Inferior STEMI and Complete Heart Block During PTMC in Pregnancy Balloon catheter manipulation during PTMC can lead to dislodgement of left atrial thrombus, resulting in coronary embolism and acute right coronary artery occlusion, presenting with inferior STEMI and complete heart block. A stentless percutaneous coronary intervention strategy, comprising balloon angioplasty, aspiration thrombectomy, and intracoronary glycoprotein IIb/IIIa inhibitor bolus administration, could restore TIMI flow grade 3. Avoidance of stent implantation eliminates the need for prolonged dual antiplatelet therapy, thereby optimizing maternal and fetal safety. AV = atrioventricular; BP = blood pressure; ECG = electrocardiogram; MI = myocardial infarction; MVA = mitral valve area; PASP = pulmonary artery systolic pressure; PCI = percutaneous coronary intervention; PTCA = percutaneous transluminal coronary angioplasty; PTMC = percutaneous transvenous mitral commissurotomy; RCA = right coronary artery; STEMI = ST-segment elevation myocardial infarction.

## History of Presentation

A 33-year-old primigravida at 31 weeks' gestation presented with progressive shortness of breath associated with orthopnea for 1 month.

**Take-Home Messages**

• The case demonstrates that coronary embolism causing inferior STEMI and complete heart block is a rare, life-threatening complication of percutaneous transvenous mitral commissurotomy in pregnancy, which requires immediate recognition and intervention.
• A tailored, stentless percutaneous coronary intervention strategy with mechanical thrombectomy can successfully restore coronary flow while minimizing prolonged dual antiplatelet therapy exposure and optimizing maternal-fetal outcomes.

## Past History

There was no history of joint pain, shortness of breath, or prolonged fever during childhood suggestive of acute rheumatic fever.

## Differential diagnosis

Clinical examination revealed loud S1 and P2 heart sounds, a mid-diastolic murmur at the cardiac apex, and bilateral basal crepitations in both lung fields, which were suggestive of critical rheumatic mitral stenosis (MS) with congestive heart failure.

## Investigations

**Figure 1:**
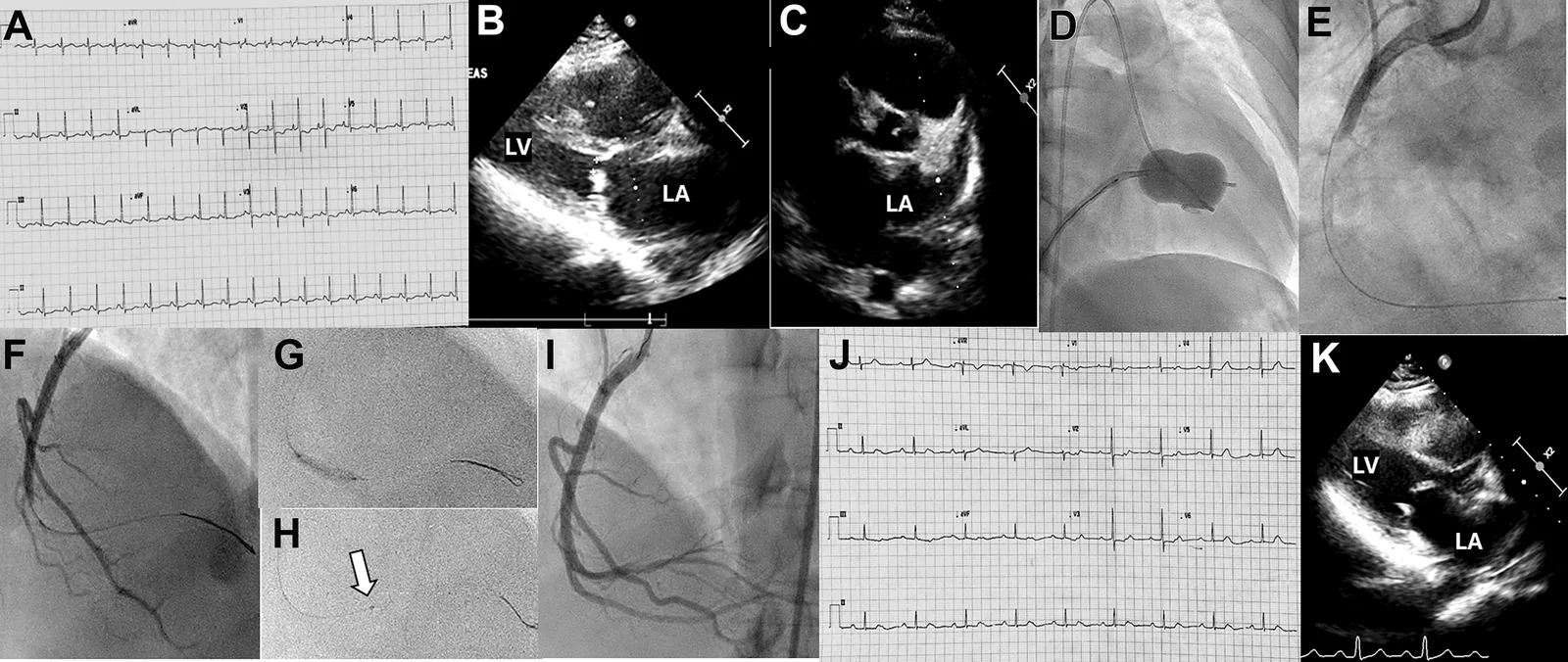
Percutaneous Transvenous Mitral Commissurotomy and Emergency Percutaneous Coronary Intervention in a Pregnant Woman (A) Baseline ECG shows sinus rhythm with left atrial enlargement and right ventricular hypertrophy, consistent with advanced mitral stenosis. (B and C) Transthoracic echocardiography shows markedly thickened, doming mitral valve leaflets producing severe mitral stenosis, with careful exclusion of left atrial and left atrial appendage thrombus. (D) Fluoroscopy confirms correct positioning and full inflation of the Inoue balloon across the mitral valve during PTMC. (E) Acute thrombotic occlusion of the mid right coronary artery with TIMI flow grade 0, and successful guidewire crossing of the culprit lesion. (F) Initial balloon angioplasty resulted in distal thrombus migration. (G) Stepwise balloon dilatation and (H, white arrow) mechanical thrombus aspiration (I) effectively restored TIMI flow grade 3 with minimal residual distal thrombus. (J) Postintervention ECG shows complete heart block with narrow QRS escape rhythm, consistent with transient ischemia of the atrioventricular conduction system. (K) Repeat echocardiography confirms successful commissurotomy with an adequately enlarged mitral valve orifice. ECG = electrocardiography; PTMC = percutaneous transvenous mitral commissurotomy.

Electrocardiogram demonstrated a sinus rhythm with left atrial enlargement and right ventricular hypertrophy (Figure 1A). Transthoracic echocardiography revealed a severely reduced mitral valve area of 0.47 cm^2^, transmitral peak and mean diastolic gradients of 35 and 22 mm Hg, respectively; moderate tricuspid regurgitation with an estimated pulmonary artery systolic pressure of 90 mm Hg, and a mitral valve Wilkins score of 7/16 (Figure 1B). No thrombus was visualized in the left atrium or left atrial appendage on transthoracic imaging (Figure 1C). Transesophageal echocardiography was deferred because of pregnancy-related considerations. Fetal Doppler assessment demonstrated symmetric intrauterine growth restriction.

## Management

After a multidisciplinary cardio-obstetrics team discussion, urgent percutaneous transvenous mitral commissurotomy (PTMC) was performed. A lead apron was wrapped circumferentially around the abdomen to avoid fetal irradiation. PTMC was performed using a 26-mm Inoue balloon via a standard transseptal antegrade approach after a 5,000 IU intravenous heparin bolus (Figure 1D, Video 1). The procedure resulted in a reduction of the end-diastolic transmitral gradient from 25 to 5 mm Hg. During the balloon withdrawal from the left atrium, the patient abruptly developed hypotension (systolic blood pressure: 60 mm Hg) and bradycardia. Despite atropine administration, intravenous fluids, and norepinephrine support, she developed acute chest pain with worsening hemodynamic stability. There was no pericardial effusion or significant mitral regurgitation on transthoracic echocardiography. Continuous cardiac rhythm monitoring revealed complete heart block (CHB) and ST-segment elevation in the inferior leads.

Emergent coronary angiography demonstrated a normal left coronary artery and a total thrombotic occlusion of the mid right coronary artery (RCA) with TIMI flow grade 0, without angiographic evidence of underlying atherosclerotic disease, consistent with embolic occlusion (Video 1). The RCA was cannulated with a Judkins right 6-F guide-catheter, and a coronary guide wire (BMW wire, Abbott Vascular) crossed the lesion (Figure 1E). After a 3 × 20 mm noncompliant balloon dilatation of the mid RCA, the thrombus migrated to the distal RCA (Figure 1F, Video 1). An intracoronary tirofiban (glycoprotein IIb/IIIa inhibitor) bolus dose of 20 mL was administered, without any infusion. Coronary angioplasty with a 3 × 20 mm balloon (Figure 1G) and aspiration thrombectomy using a thrombus aspiration catheter (Thrombuster II catheter, Kaneka Medix Corp) (Figure 1H, white arrow) were performed. TIMI flow grade 3 was achieved after multiple attempts of balloon dilatations and thromboaspiration (Figure 1I, Video 1). The patient had symptomatic and hemodynamic improvement on the table. Temporary transvenous pacing support was instituted given persistent CHB (Figure 1J). Activated clotting time at the end of the procedure was 350 seconds after a total intravenous heparin dose of 7,500 IU. Total fluoroscopy time was 19.6 minutes, and the radiation dose was 486 mGy.

## Outcome and Follow-Up

The patient was maintained on intravenous heparin infusion for 48 hours. The transvenous temporary pacing lead was removed after 48 hours, after recovery from CHB. Repeat echocardiography revealed mitral valve area of 1.9 cm^2^, a transmitral gradient of 7/4 mm Hg, and mild tricuspid regurgitation, with an estimated pulmonary artery systolic pressure of 55 mm Hg (Figure 1K). She was discharged on day 4 on aspirin monotherapy, beta-blockers, secondary rheumatic fever, and infective endocarditis prophylaxis. The subsequent antenatal course was uneventful, and she delivered vaginally at 36 weeks of gestation. The neonate weighed 2.0 kg with an Apgar score of 10/10 at 5 minutes. Both mother and child were well at the 6-month follow-up.

## Discussion

Rheumatic heart disease remains the most common cause of valvular heart disease encountered during pregnancy in low- and middle-income countries, with MS accounting for the majority of cases.^1^^,^^2^ Pregnancy-induced hemodynamic load peaks during the late second and early third trimesters and are poorly tolerated in patients with fixed left-sided valvular stenosis, such as MS and aortic stenosis.^2^^,^^3^ Consequently, severe MS is associated with a high risk of pulmonary edema, arrhythmias, thromboembolism, and adverse fetal outcomes.^2^ According to contemporary guideline-based risk stratification, patients with severe MS, elevated pulmonary artery pressures, and NYHA functional class >II are at particularly high risk of maternal cardiac events during pregnancy,^4^ as was the index case.

PTMC is the preferred intervention for symptomatic severe MS in pregnancy, offering effective and rapid hemodynamic relief with a low complication rate while avoiding the risks of open cardiac surgery.^5^, ^6^, ^7^ Systemic embolization during PTMC is uncommon, and coronary embolism represents an exceptionally rare manifestation.^6^, ^7^, ^8^ In the index case, the sudden onset of inferior ST-segment elevation myocardial infarction accompanied by CHB strongly implicates an acute coronary event. The angiographic appearance, absence of coronary atherosclerosis, and temporal relationship to left atrial instrumentation support an embolic mechanism. Potential sources include dislodgement of an occult left atrial thrombus or thrombus formation along intracardiac catheters despite adequate anticoagulation.^9^ Although transesophageal echocardiography offers superior sensitivity for detecting left atrial appendage thrombus, its use during pregnancy remains limited because of patient tolerance and procedural considerations.^10^ In our published experience of PTMC in 70 pregnant women, transthoracic echocardiographic screening without transesophageal echocardiography was preferred to exclude left atrial appendage clots.^7^ None of the patients had any systemic thromboembolic complications except for 1 patient, who had a transient ischemic attack after PTMC.^7^ We acknowledge that transesophageal echocardiography, intracardiac echocardiography, or enhanced high-resolution transthoracic imaging may be considered in selected high-risk pregnant patients, particularly those with atrial fibrillation, dense spontaneous echo contrast, prior thromboembolism, markedly enlarged left atrium, or suboptimal transthoracic windows.

Management of acute coronary embolism during pregnancy poses unique challenges. Thrombolytic therapy carries significant maternal and fetal bleeding risks and was therefore avoided.^4^ Given concerns regarding prolonged dual antiplatelet therapy (DAPT) and fetal exposure to P2Y_12_ inhibitors,^4^ a stentless strategy combining mechanical thrombectomy and low-dose intracoronary glycoprotein IIb/IIIa inhibition was preferred in the index case. This approach successfully restored coronary flow while minimizing long-term antithrombotic requirements. Chikkabasavaiah et al^8^ reported a case of left coronary artery thromboembolism during PTMC in a pregnant woman, which was managed with a stent implantation after a failed catheter-based mechanical thrombectomy. Given the successful stentless revascularization with restoration of TIMI flow grade 3, absence of atherosclerotic, residual coronary stenosis, sinus rhythm, and no documented left atrial thrombus or other established indication for long-term systemic anticoagulation, aspirin monotherapy was selected as the long-term antithrombotic strategy. In the setting of pregnancy, avoidance of prolonged DAPT or oral anticoagulation was considered important to minimize maternal bleeding risk and fetal drug exposure. Contemporary European Society of Cardiology cardio-obstetric guidelines recommend individualized antithrombotic strategies during pregnancy based on careful assessment of maternal thrombotic and bleeding risks.^4^ The presence of intrauterine growth restriction in the index case underscores the pathophysiological impact of critical MS on uteroplacental perfusion. Timely relief of mitral obstruction likely contributed to the stabilization of maternal hemodynamics and the favorable perinatal outcome.^6^^,^^7^

## Conclusions

A key highlight of this case is the successful use of a stentless mechanical thrombectomy, which enabled effective revascularization while avoiding stent implantation and the need for prolonged DAPT—an important advantage in pregnancy to minimize fetal drug exposure. This tailored, physiology-driven approach balanced maternal safety with fetal considerations. To our knowledge, this is the first reported case of a combination of acute coronary embolism presenting as an inferior ST-segment elevation myocardial infarction with complete heart block during PTMC in pregnancy, adding significant clinical relevance and novelty beyond the few isolated reports of coronary embolism during PTMC.

## Funding Support and Author Disclosures

The authors have reported that they have no relationships relevant to the contents of this paper to disclose.

## Appendix

Video 1 Percutaneous Transvenous Mitral Commissurotomy Followed by Emergency Percutaneous Coronary Intervention of an Acutely Occluded Right Coronary Artery The video demonstrates Inoue balloon–mediated mitral commissurotomy and subsequent coronary angiography, which identifies acute thrombotic occlusion of the right coronary artery, followed by balloon angioplasty and mechanical thrombus aspiration, restoring TIMI flow grade 3.
